# Relationship between coffee consumption and vasomotor symptom severity in Turkish Postmenopausal Women

**DOI:** 10.4314/ahs.v26i2.16

**Published:** 2026-06

**Authors:** Yağmur Sürmeli Akbal, Nazife Akan, Fügen Özcanarslan

**Affiliations:** 1 Harran Üniversity, Viransehir School of Health Sciences; 2 Toros Üniversity, Faculty of Health Sciences

**Keywords:** Postmenopause, Coffee consumption habits, Vasomotor symptoms

## Abstract

**Background:**

Vasomotor symptoms during menopause can be affected by many biological, psychological and environmental factors. Coffee consumption may also be one of these factors.

**Purpose:**

This study aims to investigate the relationship between coffee consumption habits and the severity of vasomotor symptoms in postmenopausal women in Turkey.

**Method:**

A cross-sectional, descriptive study was conducted with 169 postmenopausal women. Data on participants' sociodemographic characteristics, coffee consumption habits, and vasomotor symptom severity were collected. ordinal regression analyses were performed to examine the effects on symptom severity; the significance level was set at p < 0.05.

**Results:**

The mean age of participants was 57.76 ± 5.27 years, and the mean age at menopause was 49.44 ± 3.25 years. The majority of women (78.1%) consumed mainly Turkish coffee; approximately half drank 0-1 cups of coffee per day. Regression analyses revealed that coffee type and consumption temperature were significantly associated with some vasomotor symptoms.

**Conclusion:**

Coffee type and consumption temperature may be associated with the severity of some vasomotor symptoms in the postmenopausal period. Future, larger-scale studies are needed to further clarify these relationships.

## Background

Menopause is a natural biological process in women. It occurs with the cessation of the menstrual cycle^1^. Globally, it is estimated that by 2030, approximately 1.2 billion women will be in the menopausal or postmenopausal phase. Additionally, 47 million new women are expected to enter menopause each year^2^,^3^. These projections highlight the need for a more comprehensive approach to menopausal health policies today.

Menopause-related declines in estrogen levels lead to various physical (e.g., urogenital problems) and mental (e.g., depression, sleep disturbances) symptoms in women^4^,^5^. Among these symptoms, vasomotor symptoms directly impact quality of life. Characterized by facial flushing, hot flushes, and night sweats^6^, these symptoms typically persist for an average of 7.4 years^7^. The mechanism of development is explained by thermoregulatory dysfunction caused by a decrease in estrogen feedback in the hypothalamus^6^. Additionally, factors such as stress, hot beverages, alcohol, physical activity, and emotional states can also trigger symptoms^7^. A meta-analysis reported that 60-80% of women experience vasomotor symptoms during and/or after the menopausal transition^8^. A multicentre study conducted as part of an international collaboration showed that the prevalence of symptoms in women in their late 50s ranged from 30% to 50%^9^. A study conducted in Turkey reported that hot flushes and sweating were the most commonly experienced symptoms in women aged 45-60^10^.

Although the frequency of vasomotor symptoms generally decreases during the postmenopausal period, these symptoms may persist for many years in some women. In a study by Freeman and colleagues (2014), it was reported that moderate to severe hot flushes lasted an average of five years after menopause and persisted for at least 10 years in more than one-third of women^11^. A more recent study found that over 10% of Scandinavian women still experienced moderate to severe vasomotor symptoms in the postmenopausal period^12^. The duration and severity of symptoms vary depending on individual differences, hormonal levels, and lifestyle. Long-lasting symptoms during the postmenopausal period can impair quality of life, contributing to sleep problems, psychological distress, social limitations, and reduced productivity^13^.

Because vasomotor changes can negatively affect daily activities and lead to a decline in quality of life, various lifestyle and dietary recommendations have been developed to alleviate these symptoms. Among these, reducing tobacco and alcohol consumption and limiting caffeine intake have been emphasized. This may stem from the belief that caffeine worsens vasomotor symptoms. High coffee intake has been linked to earlier menopause^14^ and hormonal changes, including lower estrogen and higher progesterone levels^15^. However, studies examining the relationship between caffeine and vasomotor symptoms are limited and their results are inconsistent^16^-^18^. This highlights the need for further research to clarify the relationship between coffee consumption and vasomotor symptoms during the menopausal transition.

In this context, the aim of this study was to examine the relationship between coffee consumption habits and the severity of vasomotor symptoms among postmenopausal women in Türkiye. The results are expected to fill existing knowledge gaps and contribute to the literature in a cultural context.

## Methods

### Study design and participants

This descriptive and cross-sectional study was conducted with menopausal women living in Turkey. The relationship between coffee consumption habits and the severity of vasomotor symptoms among these women was examined. The study was carried out in accordance with the principles of the Helsinki Declaration and was approved by the Toros University Scientific Research and Publication Ethics Committee (Ethics Committee Decision No: [33], Date: [24.02.2025]). Written informed consent was obtained from all participating women.

This study was conducted between 20 March 2025 and 20 July 2025 with 169 postmenopausal women residing in Turkey. Participants were identified using the snowball sampling method. The questionnaire was first sent to participants selected by the researchers, who were then asked to forward it to other suitable participants within their social circles. The questionnaire was also shared on various social media platforms to reach women in the menopausal period.

The women's menopausal status was determined by a doctor's diagnosis of menopause following a medical evaluation. Only women who had entered menopause, had access to and could use technological devices, were literate in Turkish, were willing to participate in the study, and gave their consent were included in the study. Conversely, women who had not been diagnosed as having entered menopause by a doctor; those without access to or unable to use devices, those who did not speak Turkish, those unwilling to participate in the study, those who did not give consent, or those with psychiatric/communication issues were excluded from the study.

### Data Collection

Data were collected online using a Descriptive Information Form, a Vasomotor Symptoms and Severity Form^19^-^21^, and a Coffee Consumption Habits Form^9^,^22^,^23^ all developed by the researchers based on relevant literature. A pilot test with 10 participants confirmed the clarity of the forms; therefore, no revisions were made, and their responses were included in the study. Data were collected online via Google Forms, and at the beginning of the survey, the statement “I have read the above information and voluntarily agree to participate in the study” was provided to obtain informed consent. Only the data of participants who gave consent were included in the analysis.

### Outcome measures

The data collection tools created by the researchers in line with the literature are as follows. The Descriptive Information Form consists of 11 questions covering age, marital status, education, employment, income level, and the manner of entering menopause. The Vasomotor Symptoms and Severity Form was developed by the researcher based on studies in the literature^19^-^21^. The form includes questions to assess the type, severity, and duration of common vasomotor symptoms (hot flashes, night sweats, excessive sweating, palpitations, and headaches) during menopause. This form is not a standard scale that has undergone previous validity and reliability studies. Symptom severity is classified as mild, moderate, and severe; mild symptoms are defined as those that do not affect daily life, moderate symptoms as those that partially affect daily activities, and severe symptoms as those that significantly affect daily life. The Coffee Consumption Habits Form was developed by the researchers based on relevant literature^9^,^22^,^23^ and includes nine questions addressing coffee type, daily consumption amount, consumption style, and factors influencing coffee preferences.

### Statistical analysis

The data were analysed using IBM SPSS Statistics for Windows, version 25.0 (IBM Corp., Armonk, NY, USA). Descriptive statistics were presented as frequency, percentage, mean, and standard deviation. The normality of numerical variables was assessed using the Kolmogorov–Smirnov test. Ordinal regression analysis was performed because the dependent variables (symptom severity levels) were ordinal in nature (mild, moderate, severe). This model is appropriate for analysing ordered categorical outcomes while controlling for multiple independent variables. Hot flushes, night sweats, excessive sweating, palpitations, and headache severity were used as dependent variables, while demographic characteristics and coffee consumption habits were used as independent variables. Regression results were reported as B (unstandardised coefficient), SE (standard error), Wald chi-square test, Exp(B)-Odds ratio, and p-value. All analyses considered p < 0.05 as statistically significant.

## Results

**Figure 1:**
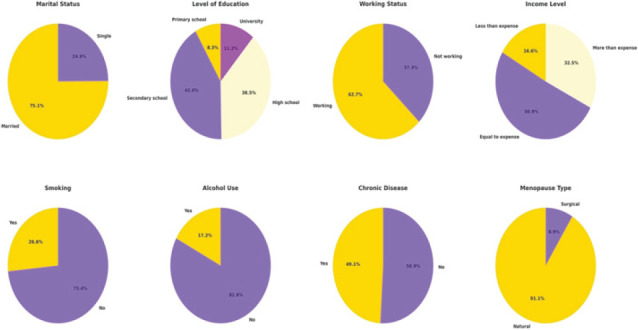
Demographic Charts

Figure 1 presents certain characteristics of the women. The mean age was 57.76 ± 5.27 years, mean age at menopause onset was 49.44± 3.25 years, and mean BMI was 26.84±4.64. Most participants were married (75.1%), had completed secondary school education (42%), were employed (62.7%), and reported balanced income and expenses (50.9%). Smoking (26.6%) and alcohol consumption (17.2%) rates were low, while 49.1% had chronic illnesses. Additionally, 91.1% experienced natural menopause.

When examining the distribution of vasomotor symptoms in postmenopausal women, 30.8% of participants reported mild hot flushes, 20.1% moderate, and 7.7% severe, while 41.4% did not experience this symptom. The mean duration of hot flushes was 13.33 ± 18.33 months. Night sweats were mild in 31.4% of participants, moderate in 20.7%, and severe in 6.5%, whereas 41.4% did not experience this symptom; the mean duration was 14.50 ± 17.23 months. Excessive sweating was reported as mild (26.0%), moderate (19.5%), and severe (10.7%), while 43.8% reported no symptoms; the mean duration was 13.82 ± 16.24 months. Palpitations were mild (33.7%) or moderate (19.5%), with no severe cases and 46.8% reporting none; the mean duration was 8.18 ± 10.45 months. Headaches were mild in 26.0% and moderate in 21.3%, with no severe headaches reported; 52.7% did not experience this symptom, and the mean duration was 8.29 ± 10.23 months.

Table 1 presents the coffee consumption habits of the women. The majority of participants (78.1%) primarily consumed Turkish coffee, approximately half (48.5%) drank 0-1 cups of coffee per day, and the most common preference was for black coffee (33.7%). Taste was identified as an important factor influencing coffee choices (67.5%). Most participants (83.4%) consumed their coffee hot, and 57.4% drank coffee at home. Additionally, 39.6% consumed coffee socially with friends, 45.6% consumed it for pleasure, and 28.4% reported that their coffee preference varied according to their mood.

**Table 1:** Distribution of women's coffee consumption habits

Category	n	%
Most Frequently Consumed Type of Coffee		
Turkish coffee	132	78.1
Filter coffee	13	7.7
Granulated Coffee	12	7.1
Other (Espresso, Latte. Americano, Mocha)	12	7.1

Daily coffee consumption		
0-1 cup	82	48.5
2-3 cups	66	39.1
4 and above	21	12.4

The most common way to consume coffee		
Plain	57	33.7
With Sugar	51	30.2
Without Sugar	36	21.3
Other (Milk, Flavored, Creamy. etc.)	25	14.8

Factor affecting coffee preference		
Taste	114	67.5
Habit	16	9.4
Social environment	13	7.7
Other (Caffeine effect. Coffee price)	26	15.1

Coffee consumption temperature		
Hot	141	83.4
Cold	28	16.6

Coffee consumption place		
At home	97	57.4
At work	40	23.7
In a friend/family member's	32	18.9

Individual(s) Consuming Coffee		
By myself	56	33.1
With friends	67	39.6
With family members (spouse, children, etc.))	46	27.2

The meaning of consuming coffee		
Enjoyable	77	45.6
Relieves stress	22	13.0
Helps with headaches	18	10.7
Energizes	19	11.2
Other (Habit forming, Social activity)	15	8.8
No point	18	10.7

Coffee preference changes depending on mood		
Yes	48	28.4
No	121	71.6

Table 2 presents the multivariate analysis of the relationship between women's sociodemographic characteristics and the severity of vasomotor symptoms. The analysis showed that palpitations were significantly associated with the 51-60 age group (p = 0.043), while night sweats demonstrated a trend toward significance (p = 0.063). Smoking was significantly associated with night sweats (p = 0.041) and excessive sweating (p = 0.011). Furthermore, alcohol consumption was significantly associated with night sweats (p = 0.044) and excessive sweating (p = 0.045), and chronic disease was significantly associated with palpitations (p = 0.025). No significant associations were found between other variables (age at menopause, BMI, educational status, employment status, income, type of menopause, marital status) and vasomotor symptoms (p > 0.05).

**Table 2:** Multivariate analysis of vasomotor symptoms by sociodemographic factors in menopausal women (n=169)

Category	Hot Flashes	Night Sweats	Excessive Sweating	Palpitations	Headaches
	B (SE)/Wald/p/Exp (B)	B (SE)/Wald/p/Exp (B)	B (SE)/Wald/p/Exp (B)	B (SE)/Wald/p/Exp (B)	B (SE)/Wald/p/Exp (B)
Age group					
(ref: 61 yaş ve üzeri)					
40-50 yaş	-0.705 (0.621) / 1.289 / 0.256 / 0.494	0.364 (0.631) / 0.332 / 0.564 / 1.439	0.414 (0.632) / 0.429 / 0.512 / 1.513	0.294 (0.671) / 0.192 / 0.661 / 1.342	0.418 (0.644) / 0.420 / 0.517 / 1.519
51-60 yaş	-0.401 (0.334) / 1.440 0.230 / 0.670	0.635 (0.341) / 3.464 / 0.063 / 1.887	0.483 (0.337) / 2.052 / 0.152 / 1.621	0.667 (0.358) / 3.462 / 0.043 / 1.949	0.154 (0.355) / 0.188 / 0.664 / 1.166

Menopause age (ref: >50)					
≤45	0.386 (0.463) / 0.694 0.405 / 1.471	0.109 (0.465) / 0.055 / 0.814 / 1.115	-0.174 (0.467) / 0.139 / 0.710 / 0.840	-0.955 (0.511) / 3.488 / 0.062 / 0.385	0.028 (0.495) / 0.003 / 0.954 / 1.028
46-50	0.575 (0.347) / 2.745 0.098 / 1.778	-0.637 (0.354) / 3.230 **/** 0.072 / 0.529	-0.467 (0.349) / 1.790 / 0.181 / 0.627	-0.647 (0.365) / 3.144 / 0.076 / 0.524	-0.002 (0.366) / 0.000 / 0.996 / 0.998

BMI group (ref: ≥30)					
<25	0.162 (1.921) / 0.007 / 0.933 / 1.176	-0.106 (0.404) / 0.435 / 0.540 / 0.055	-0.389 (0.401) / 0.830 / 0.054 / 0.789	-0.334 (0.413) / 0.734 / 0.428 / 0.308	-0.195 (**0**.412) / 0.806 / 0.640 / 0.418
25-29.9	0.189 (1.926) / 0.010 / 0.922 / 1.208	-0.112 (0.388) / 0.164 / 0.068 / 0.047	-0.387 (0.384) / 0.910 / 0.245 / 0.809	-0.917 (0.407) / 1.358 / 0.625 / 0.408	-0.497 (0.402) / 0.464 / 0.520 / 0.528

Marital Status (ref: Mairried)					
Single	-0.473 (0.357) / 1.759 / / 0.185 / 0.623	0.440 (0.366) / 1.451 / 0.228 / 1.553	0.162 (0.359) / 0.205 / 0.651 / 1.176	0.887 (0.392) / 5.116 / 0.024 / 2.428	0.916 (0.398) / 5.300 / 0.021 / 2.499

Education (ref: University)					
Primary school	0.130 (0.675) / 0.037 / 0.848 / 0.878	0.112 (0.704) / 0.025 / 0.873 / 1.119	0.496 (0.516) / 0.923 / 0.337 / 1.642	0.472 (0.731) / 0.416 / 0.519 / 1.603	-0.258 (0.734) / 0.124 / 0.725 / 0.772
Secondary school	0.026 (0.508) / 0.003 / 0.960 / 1.026	0.722 (0.523) / 1.908 / 0.167 / 2.059	0.343 (0.521) / 0.433 / 0.510 / 1.409	0.228 (0.552) / 0.171 / 0.679 / 1.256	-0.184 (0.533) / 0.119 / 0.730 / 0.832
High school	0.061 (0.512) / 0.014 / 0.905 / 1.063	0.302 (0.526) / 0.328 / 0.567 / 1.353	0.630 (0.681) / 0.856 / 0.355 / 1.878	0.811 (0.555) / 2.134 / 0.144 / 2.250	0.435 (0.533) / 0.665 / 0.415 / 1.545

Working Status (ref: Not working)					
Working	0.304 (0.331) / 0.843 / 0.359 / 1.355	-0.313 (0.336) / 0.870 / 0.351 / 0.731	-0.549 (0.333) / 2.727 / 0.099 / 0.578	0.187 (0.356) / 0.277 / 0.598 / 1.206	0.234 (0.357) / 0.429 / 0.513 / 1.264

Income (ref: More than expense)					
Less than expense	-0.609 (0.476) / 1.634 / 0.201 / 0.544	0.393 (0.483) / 0.663 / 0.416 / 1.482	-0.103 (0.480) / 0.046 / 0.830 / 0.902	0.505 (0.499) / 1.026 / 0.311 / 1.657	-0.140 (0.499) / 0.079 / 0.778 / 0.869
Equal to expense	-0.248 (0.353) / 0.492 0.483 / 0.780	0.532 (0.365) / 2.128 / 0.145 / 1.702	0.115 (0.358) / 0.103 / 0.748 / 1.122	0.041 (0.374) / 0.012 / 0.914 / 1.042	-0.420 (0.372) / 1.271 / 0.260 / 0.657

Smoking (ref: No)					
Yes	-0.136 (0.361) / 0.142 0.706 / 0.872	0.752 (0.367) / 4.195 / **0.041** / 2.121	0.926 (0.365) / 6.424 / **0.011** / 2.525	0.565 (0.382) / 2.184 / 0.139 / 1.759	0.699 (0.378) / 3.408 / **0.035** / 2.012

Alcohol Use (ref: No)					
Yes	0.189 (0.432) / 0.191 / 0.662 / 1.208	-0.589 (0.443) / 2.766 / 0.044 / 0.455	0.914 (0.456) / 4.017 / 0.045 / 0.401	-0.860 (0.478) / 3.243 / 0.072 / 0.423	-0.244 (0.459) / 0.283 / 0.045 0.783

Chronic Disease (ref: No)					
Yes	-0.097 (0.313) / 0.097 / 0.756 / 0.907	0.059 (0.318) / 0.034 / 0.854 / 1.061	0.066 (0.315) / 0.044 / 0.834 / 0.936	0.755 (0.337) / 5.027 / 0.025 / 2.128	0.523 (0.335) / 2.441 / 0.118 1.687

Menopause Type (ref: Natural)					
Surgical	0.248 (0.535) / 0.215 / 0.643 / 1.281	0.409 (0.559) / 0.537 / 0.464 / 1.505	0.238 (0.552) / 0.186 / 0.667 / 1.269	0.411 (0.563) / 0.532 / 0.466 / 1.508	0.256 (0.555) / 0.212 / 0.645 1.292

Note: B = Unstandardized regression coefficient, representing the change in the dependent variable for a one-unit change in the predictor; SE = Standard Error, indicating the variability of the coefficient estimate; Wald = Wald chi-square test statistic used to test the significance of each predictor; p = Probability value assessing statistical significance (p < 0.05 considered statistically significant); Exp(B) = Odds Ratio, representing the exponentiated value of B and showing the multiplicative change in the odds of the dependent variable associated with a one-unit change in the predictor.

Table 3 presents the multivariate relationships between women's coffee consumption habits and the severity of vasomotor symptoms. Analyses revealed a significant association between unsweetened coffee consumption and excessive sweating (p = 0.042). Significant relationships were also found between sweetened coffee consumption and hot flushes (p = 0.015), as well as Turkish coffee consumption and hot flushes (p = 0.046). No significant relationship was found between other coffee consumption variables (daily amount, filter or granulated coffee preferences, coffee consumption method, and temperature) and vasomotor symptoms (p > 0.05).

**Table 3:** Multivariate analysis of vasomotor symptoms by coffee consumption in menopausal women (n=169)

Category	Hot Flashes	Night Sweats	Excessive Sweating	Palpitations	Headaches
	B (SE)/Wald/p/Exp (B)	B (SE)/Wald/p/Exp (B)	B (SE)/Wald/p/Exp (B)	B (SE)/Wald/p/Exp (B)	B (SE)/Wald/p/Exp (B)
Most Frequently Consumed Coffee Type (ref: Other)					
Turkish coffee	1.173 (0.587) / 3.990 / **0.046** / 3.231	-0.609 (0.554) / 1.207 / 0.272 / 0.544	-0.297 (0.609) / 0.238 / 0.626 / 0.743	-0.439 (0.666) / 0.434 / 0.510 / 0.645	0.254 (0.626) / 0.164 / 0.685 / 1.290
Filter coffee	0.988 (0.797) / 1.538 / 0.215 / 2.686	0.230 (0.765) / 0.091 / 0.764 / 1.259	1.132 (0.825) / 1.880 / 0.170 / 3.101	1.483 (0.910) / 2.657 / 0.103 / 4.407	1.187 (0.863) / 1.890 / 0.169 / 3.278
Granulated Coffee	0.456 (0.792) / 0.332 / 0.564 / 1.578	-0.652 (0.774) / 0.710 / 0.400 / 0.521	-0.354 (0.861) / 0.169 / 0.681 / 0.702	-0.327 (0.942) / 0.120 / 0.729 / 0.721	0.426 (0.877) / 0.236 / 0.627 / 1.531

Daily Coffee Consumption (ref: 4 cup and above)					
0-1 cups	0.172 (0.464) / 0.137 / 0.711 / 1.188	-0.593 (0.457) / 1.680 / 0.195 / 0.553	-0.611 (0.511) / 1.430 / 0.232 / 0.543	0.220 (0.563) / 0.153 / 0.696 / 1.246	-0.292 (0.515) / 0.321 / 0.571 / 0.747
2-3 cups	-0.001 (0.472) / 0.000 / 0.999 / 0.999	-0.299 (0.464) / 0.415 / 0.519 / 0.742	-0.446 (0.523) / 0.728 / 0.394 / 0.640	0.096 (0.575) / 0.028 / 0.868 / 1.101	0.006 (0.515) / 0.000 / 0.991 / 1.006

Most Common Way to Consume Coffee (ref: Other)					
Plain	-0.760 (0.430) / 3.128 / 0.077 / 0.467	0.239 (0.415) / 0.331 / 0.565 / 1.270	0.127 (0.480) / 0.070 / 0.792 / 1.135	0.230 (0.518) / 0.198 / 0.656 / 1.259	0.539 (0.534) / 1.019 / 0.313 / 1.715
With Sugar	-1.508 (0.618) / 5.954 / **0.015** / 0.221	0.802 (0.594) / 1.822 / 0.177 / 2.231	0.991 (0.672) / 2.177 / 0.140 / 2.695	0.727 (0.737) / 0.973 / 0.324 / 2.070	0.572 (0.529) / 1.170 / 0.279 / 1.772
Without Sugar	-0.617 (0.691) / 0.798 / 0.372 / 0.540	0.372 (0.680) / 0.300 / 0.584 / 1.451	1.495 (0.762) / 3.849 / 0.062 **/** 4.457	-0.657 (0.906) / 0.526 / 0.468 / 0.519	0.471 (0.576) / 0.667 / 0.414 / 1.602

Coffee consumption temperature (ref: Cold)					
Hot	0.418 (0.387) / 1.168 / 0.280 / 1.519	0.661 (0.404) / 2.675 / 0.102 / 1.937	0.061 (0.430) / 0.020 / 0.888 / 1.063	0.627 (0.480) / 1.710 / 0.191 / 1.872	0,329 (0.438) / 0.562 / 0.453 / 1390

Note: B = Unstandardized regression coefficient, representing the change in the dependent variable for a one-unit change in the predictor; SE = Standard Error, indicating the variability of the coefficient estimate; Wald = Wald chi-square test statistic used to test the significance of each predictor; p = Probability value assessing statistical significance (p < 0.05 considered statistically significant); Exp(B) = Odds Ratio, representing the exponentiated value of B and showing the multiplicative change in the odds of the dependent variable associated with a one-unit change in the predictor.

## Discussion

This study provides important findings evaluating the relationship between coffee consumption habits and the severity of vasomotor symptoms in menopausal women. The results were interpreted in comparison with the existing literature and previous studies.

In our study, the most commonly reported vasomotor symptoms among women in menopause were hot flushes night sweats and excessive sweating. Palpitations and headaches, on the other hand, were experienced less frequently and generally of mild to moderate severity compared to other symptoms. This finding is consistent with Espitia-De La Hoz's (2022) study conducted in Colombia with 594 menopausal women. In that study, 78.8% of women reported hot flushes, while only 9.93% reported palpitations^24^. Agrali et al. (2022) also reached similar conclusions in their study 25. The prominence of these symptoms may be related to impaired thermoregulation associated with decreased estrogen levels. The lower frequency of palpitations and headaches may reflect their weaker association with menopause and greater influence from individual or environmental factors.

When the duration of vasomotor symptoms was examined, night sweats (14.50±17.23 months), excessive sweating (13.82±16.24 months), and hot flushes (13.33±18.33 months) were among the longest-lasting symptoms. These findings are consistent with the report of the American College of Obstetricians and Gynecologists (2014), which states that hot flushes typically last between 6 months and 2 years^26^. However, some studies such as those by Kingsberg et al. (2023) and Freeman et al. (2014) have indicated that these symptoms may persist for a longer duration^27^,^11^. This discrepancy may stem from factors such as demographic characteristics of the sample, cultural and climatic influences, and methodological differences.

It was determined that the most preferred type of coffee among women was Turkish coffee, and that the most influential factor in their preference was taste. A total of 56.2% of the participants reported consuming two or more cups of coffee per day. It was found that coffee was mostly consumed at home, alone, and regardless of mood (Table 1). These findings indicate that coffee has become a cultural habit embedded in daily life in Turkey, with a strong tendency toward individual consumption. There are no studies in the literature specifically addressing coffee consumption habits among menopausal women in Turkey. Only Ahmadieh and Jradi (2021) reported that more than half of menopausal women in Lebanon consumed more than three cups of coffee per day.^28^ The findings of this study represent one of the few studies conducted on this topic in Türkiye and are believed to fill a gap in the existing literature.

In our study, it was demonstrated that women aged 51-60 were at a significantly higher risk of experiencing palpitations compared to those aged 61 and above. In the literature, Zhenghua et al. (2025) similarly reported that severe symptoms began to increase among women over the age of 48, with prevalence peaking at around 56 years^29^. It is also well established that smoking, caffeine, alcohol, fatty and spicy foods, as well as environmental factors, are considered potential triggering factors for the intensification of hot flushes^30^.

In our study, smoking was found to be positively and significantly associated with the severity of night sweats and excessive sweating, whereas alcohol consumption showed a significant negative association with the same symptoms. Consistent with our findings, previous research has reported that smoking is linked to an increased risk of hot flushes and night sweats 31,32; however, some studies have indicated no association^33^ or even a negative relationship between smoking and palpitations^34^. Furthermore, while certain studies support our finding regarding alcohol consumption^35^, others have reported contrary results, suggesting a positive association between alcohol use and these symptoms^36^, or no significant relationship at all^32^,^37^. In our study, the presence of chronic diseases was found to be significantly associated with the severity of palpitations in the postmenopausal period (p=0.025). Similarly, the literature reports that chronic diseases may increase the severity of menopausal symptoms. This is because estrogen plays an important regulatory role in the functioning of the endocrine, cardiovascular, skeletal, and metabolic systems^38^. Chronic conditions such as cardiovascular disease and hypertension, combined with declining estrogen levels, may increase susceptibility to palpitations and tachycardia 32,39.

In our study, hot flush severity was significantly associated with Turkish coffee consumption (p = 0.046). No study containing specific findings according to coffee types has been found in the literature, which again emphasises the originality of our study. The literature contains findings suggesting that consumption of caffeinated beverages may be associated with the severity of menopausal symptoms; however, these studies are not recent. These findings highlight the need for further research in this area. Faubion and colleagues (2015) reported in their study of 1,806 women that caffeine consumers had higher vasomotor symptom severity (hot flushes and night sweats)^18^. Another study conducted in 2010 also showed a positive relationship between caffeinated beverage intake and hot flush complaints^16^. However, when examining some studies conducted after 2010, there is no significant relationship between caffeine consumption and vasomotor symptoms^39^, and there is a study that concludes that there are positive effects^17^. The interaction between caffeine and estrogen occurs via the same enzyme (cytochrome P450 1A2). Competition for the same enzyme may result in different physiological responses in the body. This interaction can alter the metabolism of both compounds, and this situation may vary between individuals^40^. These conflicting results can be explained by this literature information, as well as by individual differences, the type of coffee consumed, and the amount consumed. In addition, our findings provide a unique contribution to the literature by revealing that high-intensity coffee types, such as Turkish coffee, may be associated with greater symptom severity. However, due to the cross-sectional design of the study, causal inferences cannot be drawn. Additionally, although coffee temperature was examined, no significant association was found with vasomotor symptom severity.

### Limitations and future directions

The strengths of this study are as follows: Our research is robust in that it focuses on postmenopausal women, examining a specific and relevant population. The evaluation of multiple variables such as demographic characteristics, coffee consumption habits, and vasomotor symptoms within the same study broadens the scope of the findings and allows for the simultaneous analysis of different factors. The ordinal regression analysis used made it possible to evaluate the potential effects of independent variables on symptoms in a controlled manner and minimised potential confounders. Furthermore, the results obtained provide applicable information for postmenopausal women's health by revealing the possible relationships between coffee consumption and vasomotor symptoms.

The limitations of this study are as follows: The average age of our sample being approximately 58 years and the average time since menopause being eight years resulted in approximately 40% of participants not experiencing any vasomotor symptoms. This is a factor that should be considered when interpreting the findings and may limit the generalisability to women of specific ages and menopause durations. In addition factors such as the socio-economic status, educational level, and health status of the sample may also influence the frequency of symptoms. Furthermore, the use of snowball sampling and online data collection may have introduced selection bias, as participation was limited to women with internet access and adequate digital literacy, potentially reducing the representativeness of the sample. As the data were self-reported, social desirability bias cannot be excluded. Moreover, the cross-sectional design precludes causal inferences.

Recommendations for future studies are as follows:

The findings from our study indicate that the relationship between coffee consumption and menopausal symptoms requires more comprehensive evaluation. Studies that examine hormone levels, are larger in scale, and include different age groups may enable a more comprehensive and definitive assessment of these relationships. Studies including diverse socio-economic and cultural groups may enhance the generalisability of the findings and inform practical recommendations for women's health.

### Clinical implications

The findings suggest that coffee consumption may influence the severity of vasomotor symptoms. Therefore, health professionals should take coffee consumption into account when providing lifestyle counselling for women undergoing menopause and offer individualized guidance tailored to personal differences.
